# Optimizing Premenopausal Hormone Receptor-Positive Human Epidermal Growth Factor Receptor 2-Negative Early Breast Cancer Management in India: Insights From Expert Consensus

**DOI:** 10.7759/cureus.76392

**Published:** 2024-12-25

**Authors:** Ashok K Vaid, Olivia Pagani, Anita Ramesh, Anubha Bharthuar, Chirag Desai, Ghanashyam Biswas, Jyoti Wadhwa, Prabrajya N Mohapatra, Seema Gulia, SVSS Prasad, Tarini P Sahoo, Vijay Agarwal, Rohit R Desai, Bhavesh P Kotak, Femina Dawer

**Affiliations:** 1 Department of Medical Oncology, Medanta Cancer Institute, Gurugram, IND; 2 Department of Medical Oncology, Interdisciplinary Cancer Service, Hôpital Riviera-Chablais, Vaud, CHE; 3 Department of Medical Oncology, Kauvery Hospital, Chennai, IND; 4 Department of Medical Oncological Sciences and Hematology, Patel Hospital, Jalandhar , IND; 5 Department of Medical Oncology, Hemato Oncology Clinic, Vedanta Hospital, Ahmedabad, IND; 6 Department of Medical Oncology, Sparsh Hospital and Critical Care, Bhubaneswar, IND; 7 Department of Medical Oncology, Paras Healthcare, Gurugram, IND; 8 Department of Medical Oncology, Apollo Cancer Center, Kolkata, IND; 9 Department of Medical Oncology, Tata Memorial Hospital, Mumbai, IND; 10 Department of Hematology, Apollo Hospitals, Hyderabad, Hyderabad, IND; 11 Department of Medical Oncology, Silverline Hospital, Bhopal, IND; 12 Department of Medical Oncology, Apollo Hospital, Bengaluru, IND; 13 Department of Medical Affairs, Dr. Reddy's Laboratories, Hyderabad, IND

**Keywords:** chemotherapy, ovarian function suppression, premenopausal women, risk stratification, triptorelin

## Abstract

This research aims to optimize adjuvant ovarian function suppression (OFS) for premenopausal Indian women with hormone receptor-positive (HR+) /human epidermal growth factor receptor 2-negative (HER2-) early breast cancer (eBC). To address specific challenges identified in clinical practice, a comprehensive questionnaire consisting of 21 statements was developed. These statements were reviewed and validated by a scientific committee, ensuring their accuracy and relevance to the study's objectives. A panel of 46 Indian experts and one global expert in the field of eBC were asked to rate their level of agreement/disagreement with each statement. Consensus was defined as achieving ≥80% agreement among participants. Following two rounds of the modified Delphi technique, a consensus was achieved on 19 out of 21 statements addressing critical aspects of premenopausal HR+ HER2- eBC management. The expert panel strongly recommended comprehensive risk stratification for premenopausal patients with HR+ HER2- eBC, highlighting age ≤40 as a high-risk factor and advising composite assessments for patients ≥40 years. For high-risk patients, OFS coupled with an aromatase inhibitor emerged as the recommended therapeutic strategy. The panel recommended a potential duration of up to five years for OFS, provided tolerability is maintained. For patients under 40, simultaneous OFS and chemotherapy is advised when needed. For those over 40, sequential initiation is acceptable. Triptorelin is preferred among luteinizing hormone-releasing hormone analogs, though all options have similar efficacies. The outcomes of this consensus offer valuable clinical guidance, enabling individualized and evidence-based approaches for OFS in Indian patients with HR+ HER2- eBC.

## Introduction and background

Breast cancer (BC) is the most common cause of cancer-related morbidity and mortality in women worldwide [1]. According to Global Cancer Observatory 2022, BC accounted for a staggering 26.7% of all cancer cases reported globally. The estimated incidence was 2.3 million [2]. In India, the incidence of BC accounted for 13.6% of all cancer cases in 2022 [2,3]. Notably, BC is no longer confined to postmenopausal women, as it is frequently diagnosed in premenopausal women; roughly 20% of all BC cases are diagnosed in women aged <50 years [4]. Over the last 25 years, there has been a concerning rise in cases among women aged 25-40 years [5].

It is crucial to recognize that BC detected in the early stages is mostly (~80%) hormone receptor-positive (HR+) and human epidermal growth factor receptor 2-negative (HER2-) [6-8]. As most of these tumors are HR+, adjuvant hormonal therapy is often recommended to reduce the risk of recurrence after radical surgery [9,10].

For decades, clinical trials on adjuvant endocrine therapy (ET) for HR+ premenopausal patients with early breast cancer (eBC) have demonstrated significant reductions in BC mortality for up to 15 years from diagnosis [11-13].

Most recently, large international trials, such as Suppression of Ovarian Function Trial (SOFT) and Tamoxifen and Exemestane Trial (TEXT), have shown that ET is associated with decreased BC recurrence rates and deaths and have revealed the benefits of adding ovarian function suppression (OFS) in patients at a higher risk of disease relapse. The extended, 12-year follow-up data of SOFT and TEXT confirm the role of OFS in this treatment. Combining luteinizing hormone‑releasing hormone analogs (LHRHa) with tamoxifen or aromatase inhibitors (AIs) has strengthened the role of OFS in the management of premenopausal patients with eBC [14-16].

Despite evidence that OFS improves overall survival (OS) in premenopausal women for at least 20 years of follow-up, its use is still contentious [17]. An ideal OFS-based regimen and course of treatment is yet to be decided [18]. Geographical variations in the epidemiology of the disease and a lack of real-world evidence in Indian settings warrant a national consensus guideline for OFS indications.

The benefits of chemotherapy (CT) depend on tumor biology, menopausal status, and risk features such as genomic scores or Ki-67 [19]. As per the European Society for Medical Oncology (ESMO) clinical guidelines on adjuvant therapy for HR-positive, HER2-negative eBC, lower risk cases are generally managed with tamoxifen or an AI for five years, while higher risk cases prioritize AI, extended treatment durations, and OFS, particularly in women under 35 [19].

BC in Asia presents unique challenges, with a higher proportion of patients diagnosed with de novo metastatic disease, locally advanced cases, and multiple involved sites compared to Western countries. Guidelines, such as those from ESMO, the American Society of Clinical Oncology (ASCO), and the National Comprehensive Cancer Network (NCCN), present conflicting recommendations for prognostic tests for lymph node-negative and node-positive eBC. The use of Western guidelines, designed primarily for the Caucasian population, can result in inappropriate overtreatment or undertreatment in Asia and India. Without appropriate risk assessment, patients might be unnecessarily exposed to CT and its risks. Resource limitations also hinder optimal care for these patients [20,21].

Controversies on risk-stratification evaluation, usage of adjuvant ET, and the role of OFS in the management of premenopausal HR+ HER2- eBC led us to conduct a Delphi survey to collect expert opinions and reach a consensus on the current treatment approaches to help optimize the management of premenopausal HR+ HER2- eBC in India. This manuscript’s abstract was previously presented as a poster at ESMO Asia Singapore 2023 on December 2, 2024.

Need for an Indian consensus for optimizing the management of premenopausal HR+ HER2- eBC

The burden of BC in India emphasizes the endemicity of the problem. There is a need for guidelines that are specific to the Indian setting because approximately one-third of the newly diagnosed invasive BC cases occur in women aged <50 years [4,22]. Although there is evidence that OFS contributes to an OS benefit of up to 20 years of follow-up in premenopausal patients, its use is still controversial [11,12]. Hence, to address challenges in clinical practice, this consensus was carried out using the modified Delphi technique to arrive at a definition of clinical risk stratification in premenopausal patients with HR+/HER2- eBC and managing premenopausal patients with HR+/HER2- eBC.

## Review

Materials and methods

Literature Review

The core group of 12 experts conducted a comprehensive search across the PubMed and Google Scholar databases to identify articles on the management of patients with HR+/HER2-negative eBC. The studies were identified systematically using the population, intervention, control, and outcomes format, focusing on papers published between 2019 and 2024. Additionally, a gray literature search was also conducted to gather specific information on relevant topics. A total of 14,467 articles were screened, and 48 were selected for the development of consensus statements.

The search string is elaborated below: (((("hormon"[All Fields] OR "hormonal"[All Fields] OR "hormonally"[All Fields] OR "hormonals"[All Fields] OR "hormone s"[All Fields] OR "hormones"[Pharmacological Action] OR "hormones"[MeSH Terms] OR "hormones"[All Fields] OR "hormone"[All Fields] OR "hormons"[All Fields]) AND "receptor-positive"[All Fields]) OR (("human s"[All Fields] OR "humans"[MeSH Terms] OR "humans"[All Fields] OR "human"[All Fields]) AND ("erbb receptors"[MeSH Terms] OR ("erbb"[All Fields] AND "receptors"[All Fields]) OR "erbb receptors"[All Fields] OR ("epidermal"[All Fields] AND "growth"[All Fields] AND "factor"[All Fields] AND "receptor"[All Fields]) OR "epidermal growth factor receptor"[All Fields]) AND "2-negative"[All Fields]) OR "eBC"[All Fields] OR ("hepatoma res"[Journal] OR "hr"[All Fields]) OR "her2"[All Fields] OR "OFS"[All Fields] OR (("ovarian"[All Fields] OR "ovarians"[All Fields]) AND ("functional"[All Fields] OR "functional s"[All Fields] OR "functionalities"[All Fields] OR "functionality"[All Fields] OR "functionalization"[All Fields] OR "functionalizations"[All Fields] OR "functionalize"[All Fields] OR "functionalized"[All Fields] OR "functionalizes"[All Fields] OR "functionalizing"[All Fields] OR "functionally"[All Fields] OR "functionals"[All Fields] OR "functioned"[All Fields] OR "functioning"[All Fields] OR "functionings"[All Fields] OR "functions"[All Fields] OR "physiology"[MeSH Subheading] OR "physiology"[All Fields] OR "function"[All Fields] OR "physiology"[MeSH Terms]) AND ("suppress"[All Fields] OR "suppressed"[All Fields] OR "suppresser"[All Fields] OR "suppresses"[All Fields] OR "suppressibility"[All Fields] OR "suppressible"[All Fields] OR "suppressing"[All Fields] OR "suppression"[All Fields] OR "suppressions"[All Fields] OR "suppressive"[All Fields] OR "suppressives"[All Fields])) OR "LHRHa"[All Fields] OR ("aromatase inhibitors"[Pharmacological Action] OR "aromatase inhibitors"[MeSH Terms] OR ("aromatase"[All Fields] AND "inhibitors"[All Fields]) OR "aromatase inhibitors"[All Fields]) OR (("adjuvancy"[All Fields] OR "adjuvanted"[All Fields] OR "adjuvanting"[All Fields] OR "adjuvants"[All Fields] OR "adjuvants pharmaceutic"[Pharmacological Action] OR "adjuvants immunologic"[Pharmacological Action] OR "adjuvants, pharmaceutic"[MeSH Terms] OR ("adjuvants"[All Fields] AND "pharmaceutic"[All Fields]) OR "pharmaceutic adjuvants"[All Fields] OR "adjuvant"[All Fields] OR "adjuvants, immunologic"[MeSH Terms] OR ("adjuvants"[All Fields] AND "immunologic"[All Fields]) OR "immunologic adjuvants"[All Fields] OR "adjuvated"[All Fields] OR "adjuvation"[All Fields] OR "adjuvent"[All Fields]) AND ("etiology"[MeSH Subheading] OR "etiology"[All Fields] OR "et"[All Fields]))) AND 2018/06/01:2023/06/30[Date - Publication] AND ("Breast Neoplasms"[MeSH Terms] AND 2018/06/01:2023/06/30[Date - Publication])) AND (2018/6/1:2023/6/30[pdat]).

Delphi Questionnaire

A thorough literature review was conducted on the relevant topics, and the uncertainties in the management of patients with HR+/HER2-negative eBC were highlighted. The practical challenges in managing these patients in the Indian scenario were summarized in 21 statements by the 12 steering committee members/core panel experts. The statements were later drafted as multiple-choice questions for the survey. The two major domains of the questionnaire were 1) risk stratification in premenopausal patients with HR+/HER2-negative eBC and 2) management strategies in premenopausal patients with HR+/HER2-negative eBC.

Delphi Experts

The scientific committee was comprised of 12 oncologists with a Doctor of Medicine in Medical Oncology and 10-15 years of experience in the treatment of BC. All the experts selected for the Delphi panel were involved in providing care for patients with BC at tertiary-level hospitals, as mentioned in Appendices 1 and 2. These experts were invited to join the panel from different societies, including the ESMO, ASCO, Indian Cancer Society, and the San Antonio Breast Cancer Symposium (SABCS). The moderator of the panel and lead author of this article is an international consultant on BC treatment and has been part of several societies and expert committees (ESMO, ASCO, SABCS, and St. Gallen International Consensus Conference) on ovarian function suppression.

The committee carried out an extensive literature review to create the Delphi questionnaire, generated consensus statements, defined the consensus level and Delphi methodology, selected the Delphi expert panel, interpreted and discussed the results of the Delphi questionnaire after each round, and developed the final consensus document.

All experts who agreed to participate in the Delphi process received an electronic link for personalized access to an online voting platform for the Delphi consensus.

Two-Round Modified Delphi Process

**Figure 1:**
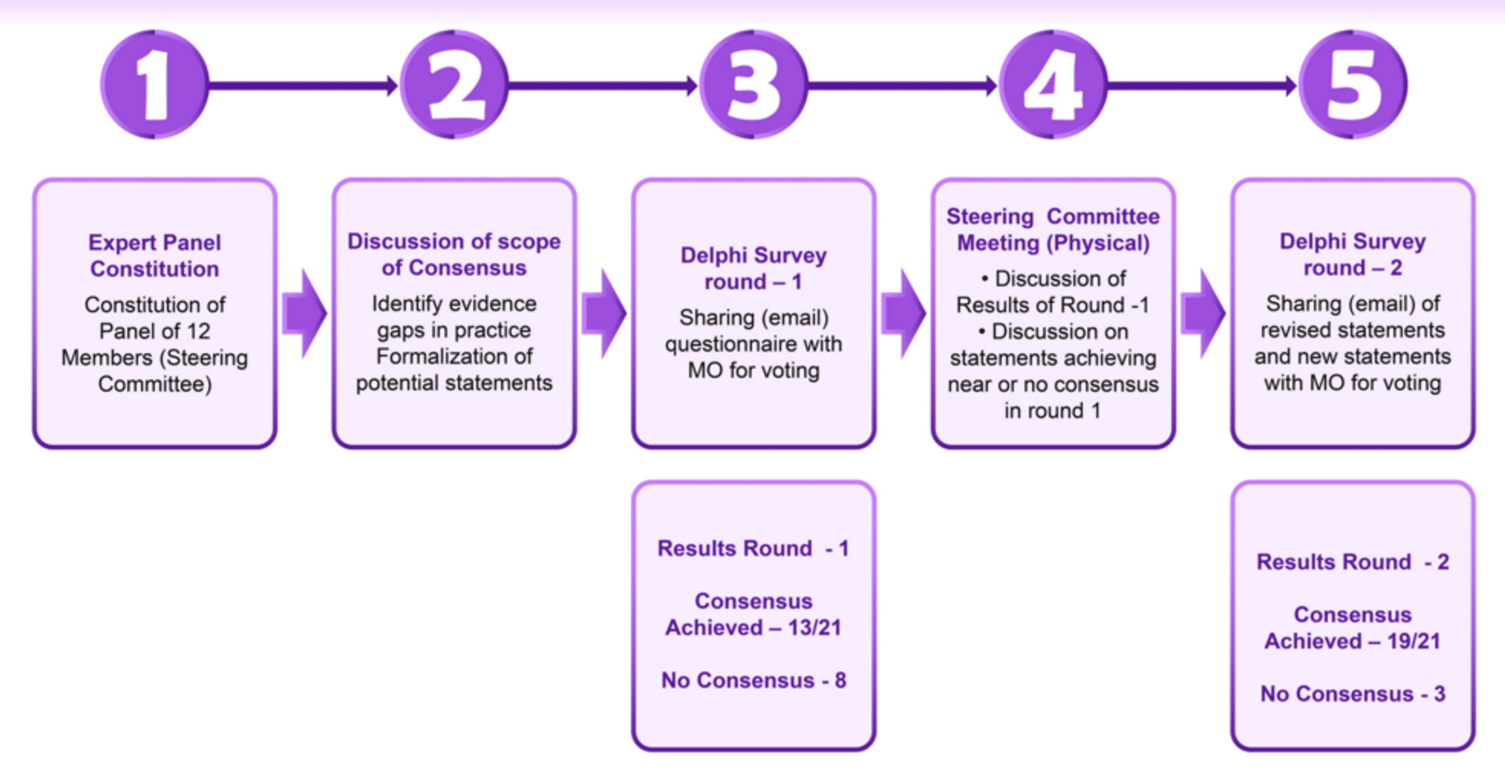
Steps in the development of the consensus statements Source: This is an original image created by the author Ashok K. Vaid

A group of 49 experts from India participated in an online survey to evaluate 21 statements during the first round of the modified Delphi process (Appendix 1). The statements were considered to have reached a high consensus when ≥80% of experts agreed upon a response. This threshold was used as many recently published studies utilizing the modified Delphi method used thresholds of >80% to reach consensus; this is in line with literature that suggested that at least 80% agreement is needed to achieve content validity in a group of 10 or more experts [23-26]. The physical meeting of the scientific steering committee (with 12 experts) formed the second round of the modified Delphi process (Appendix 2). Some of the statements were revised based on feedback from the experts during the online survey and were discussed with the experts during the steering committee meeting. During the meeting, the reasons for disagreements were highlighted, and the experts’ discussions were documented to be addressed in the consensus paper. The presentation of each recommendation was followed by a discussion (Figure 1).

Statistical Analysis

The results of both the first and second modified Delphi rounds were merged and summarized using descriptive statistics. The Delphi experts’ agreement with each statement was reported as a percentage. Consensus was achieved when >80% of experts agreed or disagreed with the statement.

Results and discussion

A total of 19 statements reached consensus after two rounds of the modified Delphi process; three statements did not reach consensus (Tables 1-4).

**Table 1:** Independent and composite risk stratification for recurrence in women with HR+/HER2- eBC eBC: early breast cancer; ER: estrogen receptor; HER2-: human epidermal growth factor receptor 2-negative; HR+: hormone receptor-positive; Ki-67: antigen Kiel 67

S. no.	Consensus statement	Agreement (%)	Consensus (yes/no)
1	Risk stratification for premenopausal patients with HR+/HER2- eBC should be performed for definitive management	98	Yes
2	Age at diagnosis (≤/≥40 years) is an important independent risk factor in classifying premenopausal patients with HR+/HER2- eBC as high risk	67	No
Utilization of genomic assays for risk stratification			
3	To identify high-risk patients, the genomic assay should be routinely offered to patients with node-negative HR+ eBC	58	No
Composite risk analysis			
4	A composite risk analysis is to be considered for a patient at high risk of recurrence based on the following criteria: node-positive 1-3, ER/progesterone receptor <50%, grade 2/3, tumor size >2 cm, and Ki-67 >20%	92	Yes

**Table 2:** OFS in premenopausal and perimenopausal women with HR+/HER2- eBC eBC: early breast cancer; ET: endocrine therapy; FSH: follicle-stimulating hormone; HER2-: human epidermal growth factor receptor 2-negative; HR+: hormone receptor-positive; LHRHa: luteinizing hormone releasing hormone analog; OFS: ovarian function suppression; RANK: receptor activator of nuclear factor-kappaB

S. no.	Consensus statement	Agreement (%)	Consensus (yes/no)
Considerations for OFS			
5	Women with high-risk HR+/HER2- eBC should be considered for OFS in addition to oral adjuvant ET	83	Yes
6	The panel recommends that OFS should not be offered to all premenopausal patients with HR+/HER2- eBC, irrespective of risk stratification	100	Yes
Duration of OFS			
7	If well tolerated, the optimal duration for OFS should be five years in premenopausal women with HR+/HER2- eBC	83	Yes
Choice of agent			
8	Although all LHRHa have similar efficacy, triptorelin is the LHRHa of choice for OFS in premenopausal women with HR+/HER2- eBC	92	Yes
Preparation of LHRHa			
9	Monthly formulation of LHRHa is preferred for ovarian suppression in premenopausal women with HR+/HER2- eBC	50	No
OFS in perimenopausal women			
10	OFS should be considered in perimenopausal women with HR+/HER2- eBC if the baseline FSH and estradiol levels are not in the postmenopausal range	100	Yes
11	Serum FSH and estradiol levels help establish menopausal status	92	Yes
12	Bisphosphonates or RANK inhibitors should be considered in all perimenopausal women who undergo OFS, either as adjuvant therapy or as a preventive strategy	85	Yes
13	OFS with oral ET therapy may benefit some perimenopausal women with eBC	91	Yes

**Table 3:** ET considerations along with OFS AI: aromatase inhibitor; CT: chemotherapy; OFS: ovarian function suppression

S. no.	Consensus statement	Agreement (%)	Consensus (yes/no)
14	AI should be preferred over tamoxifen as the backbone for OFS	92	Yes
15	Tamoxifen monotherapy can be considered for premenopausal women with a low risk of recurrence and where CT is not recommended	87	Yes
16	AI should not be offered without OFS for premenopausal women	96	Yes
17	The addition of AI, given either for five years or two to three years after two to three years of tamoxifen, results in a greater reduction in the risk of recurrence than five years of tamoxifen monotherapy	96	Yes

**Table 4:** CT considerations and treatment strategies CT: chemotherapy; eBC: early breast cancer; HER2-: human epidermal growth factor receptor 2-negative; HR+: hormone receptor-positive

S. no.	Consensus statement	Agreement (%)	Consensus (Yes/No)
18	CT should be considered in HR+/HER2- eBC premenopausal women with a high risk of recurrence based on clinicopathological assessment and/or genomic assay	96	Yes
19	Anthracycline- and taxane-based regimens should be preferred in premenopausal women with HR+/HER2- eBC	80	Yes
20	Adjuvant CT in premenopausal patients has a dual effect: an indirect endocrine and a direct cytotoxic effect	87	Yes
21	The decision to use adjuvant chemoendocrine therapy concurrently or sequentially in premenopausal women with HR+/HER2- eBC should be case-based	84	Yes

**Figure 2:**
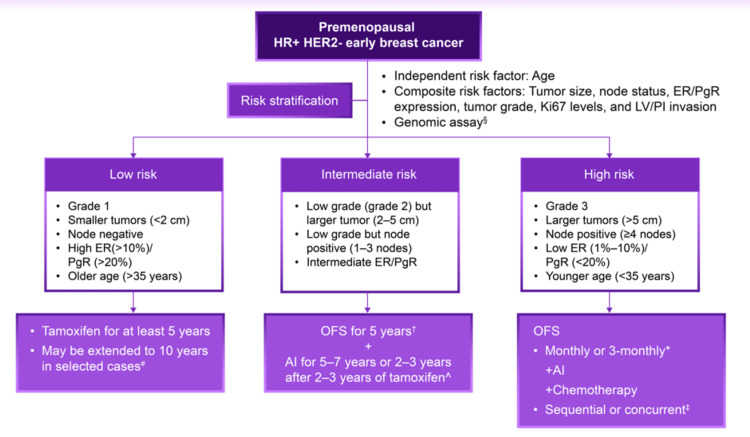
Consensus-based algorithm for the role of OFS in the treatment of HR+/HER2- eBC in premenopausal women. ^§^Offered to patients with node-negative HR+ EBC ^#^In patients with a higher stage tumor or extensive lymph node involvement (greater than or equal to four nodes) ^†^May be discontinued at two years based on treatment tolerability ^^^Based on clinical practice in selected cases by extrapolating the results of AIs in postmenopausal women ^*^Case-to-case basis with close monitoring of ovarian function for patient feasibility ^‡^Chemotherapy administered concomitantly with OFS+AI is preferred in younger women, whereas sequential initiation is preferred in older women AI: aromatase inhibitor; eBC: early-stage breast cancer; ER: estrogen receptor; HER2-: human epidermal growth factor receptor 2; HR: hormone receptor; LV/PI: lymphovascular and perineural invasion; OFS: ovarian function suppression; PgR: progesterone receptor Source: [13,34,39,48-53]

The level of evidence for each statement is provided in Table 5 [6,8,11,12,14-17,27-47]. The experts developed a treatment algorithm for the role of OFS in treating premenopausal women with HR+/HER2- eBC during a board meeting (Figure 2) [13,34,39,48-53].

**Table 5:** Consensus statements with Oxford level of evidence and grade AI: aromatase inhibitor; CT: chemotherapy; eBC: early breast cancer; ER: estrogen receptor; ET: endocrine therapy; FSH: follicle-stimulating hormone; HER2-: human epidermal growth factor receptor 2-negative; HR+: hormone receptor-positive; Ki-67: antigen Kiel 67; LHRHa: luteinizing hormone releasing hormone analog; OFS: ovarian function suppression; RANK: receptor activator of nuclear factor-kappaB Source: [6,8,11,12,14-17,27-47]

S. no.	Consensus statement	Agreement (%)	Consensus (yes/no)	Reference	Oxford level of evidence and grade
1	Risk stratification for premenopausal patients with HR+/HER2- eBC should be performed for definitive management	98	Yes	[27]	5
2	Age at diagnosis (≤/≥40 years) is an important independent risk factor in classifying premenopausal patients with HR+/HER2- eBC as high risk	67	No	[28,29]	3b 3b
Utilization of genomic assays for risk stratification					
3	To identify high-risk patients, the genomic assay should be routinely offered to patients with node-negative HR+ eBC	58	No	[6,30,31]	5 5 5 2b
Composite risk analysis					
4	A composite risk analysis is to be considered for a patient at high risk of recurrence based on the following criteria: node-positive 1-3, ER/progesterone receptor <50%, grade 2/3, tumor size >2 cm, and Ki-67 >20%	92	Yes	[15,32,33]	2b 5 5
Considerations for OFS					
5	Women with high-risk HR+/HER2- eBC should be considered for OFS in addition to oral adjuvant ET	83	Yes	[34-37]	1 1 5 1
6	The panel recommends that OFS should not be offered to all premenopausal patients with HR+/HER2- eBC, irrespective of risk stratification	100	Yes	[8]	1a
Duration of OFS					
7	If well tolerated, the optimal duration for OFS should be five years in premenopausal women with HR+/HER2- eBC	83	Yes	[27]	5
Choice of agent					
8	Although all LHRHa have similar efficacy, triptorelin is the LHRHa of choice for OFS in premenopausal women with HR+/HER2- eBC	92	Yes	[14,38]	2b 2b
Preparation of LHRHa					
9	Monthly formulation of LHRHa is preferred for ovarian suppression in premenopausal women with HR+/HER2- eBC	50	No	[39]	1b
OFS in perimenopausal women					
10	OFS should be considered in perimenopausal women with HR+/HER2- eBC if the baseline FSH and estradiol levels are not in the postmenopausal range	100	Yes	[11]	1a
11	Serum FSH and estradiol levels help establish menopausal status	92	Yes	[40]	5
12	Bisphosphonates or RANK inhibitors should be considered in all perimenopausal women who undergo OFS, either as adjuvant therapy or as a preventive strategy	85	Yes	[41]	1a
13	OFS with oral ET therapy may benefit some perimenopausal women with eBC	91	Yes	[42]	5
14	AI should be preferred over tamoxifen as the backbone for OFS	92	Yes	[11,16]	1a 1b
15	Tamoxifen monotherapy can be considered for premenopausal women with a low risk of recurrence and where CT is not recommended	87	Yes	[33,36,37,43]	1 5 5 1
16	AI should not be offered without OFS for premenopausal women	96	Yes	[11]	1a
17	The addition of AI, given either for five years or two to three years after two to three years of tamoxifen, results in a greater reduction in the risk of recurrence than five years of tamoxifen monotherapy	96	Yes	[15]	1b
18	CT should be considered in HR+/HER2- eBC premenopausal women with a high risk of recurrence based on clinicopathological assessment and/or genomic assay	96	Yes	[27,43]	1 5
19	Anthracycline- and taxane-based regimens should be preferred in premenopausal women with HR+/HER2- eBC	80	Yes	[12,27]	5 1a
20	Adjuvant CT in premenopausal patients has a dual effect: an indirect endocrine and a direct cytotoxic effect	87	Yes	[17,44]	5 5
21	The decision to use adjuvant chemoendocrine therapy concurrently or sequentially in premenopausal women with HR+/HER2- eBC should be case-based	84	Yes	[45-47]	1b

Risk Stratification

The risk of recurrence in premenopausal HR+/HER2- eBC depends on independent and composite risk factors, and several tumor and patient-related factors are part of the composite risk algorithm.

Independent and composite risk stratification: The appropriate definition of “high” and “low” risk HR+/HER2- eBC is debatable. The HER2 expression and HR+/- status are used by the NCCN guidelines to categorize patients with BC. Further, they are stratified based on anatomic and pathological parameters [43,51]. The majority of the experts (98%) concurred that risk stratification should be performed in patients with premenopausal HR+/HER2- eBC for effective management recommendations (statement no. 1) (level of evidence: 5), and this statement is supported by the 2019 St. Gallen International Consensus Guidelines [27]. Most (67%) experts recommended that only age can be considered a single independent risk factor, with the remaining components being a composite of those used in the SOFT and TEXT trials. In women aged 35 years or younger, triple-negative, HER2-positive, and luminal-B subtypes were more frequently observed. It was also noted that eBC was linked to larger tumors, higher grade, estrogen receptor (ER)-negativity, and lymph node involvement with poor prognosis [29]. The panelists opined that <40 years of age is considered high risk, whereas <35 years is very high risk (statement no.2) (level of evidence: 3b).

Utilization of genomic assays for risk stratification: The statement on whether genomic assays should be offered to patients with node-negative HR+/HER2- eBC to identify patients at high risk of disease relapse (statement no. 3) (level of evidence: 2b) was agreed upon by 58% of experts. This is in agreement with the recommendations of the 17th St. Gallen International Breast Cancer Conference in 2021 [6,30]. The experts acknowledged that although they consistently recommend a genomic assay for their patients, practical considerations like high cost and long turnaround times pose a significant problem. In such situations, only clinicopathological parameters are considered for decision-making. Panelists opined that genomic assays should not be utilized in patients who are node-positive for greater than three lymph nodes. Additionally, the experts recommended that genomic assays could help identify high-risk patients eligible for CT and OFS and intermediate-risk patients with unclear treatment options. Also, it provides information about the duration of CT. This statement is in agreement with the St. Gallen International Consensus Guidelines for treating eBC [27].

The ASCO guidelines strongly advocate genomic assays in guiding decisions regarding adjuvant CT for individuals with ER+, HER2-, and node-negative BC. Additionally, the NCCN guideline acknowledges genomic assays as the method for predicting the benefits of CT. It has been integrated into the NCCN guidelines for patients with ER+ HER2- tumors >0.5 cm and for those with pathological node-negative BC or micrometastatic nodal involvement. Experts recommend that tumors <0.5 cm do not require genomic testing; however, tumors of size 0.5-1 cm may require genomic testing depending on the presence of other high-risk factors. Genomic testing is recommended in individuals with tumors >1 cm in size, even if they are node-negative [54]. Three phase-3 studies, Microarray In Node negative Disease may Avoid ChemoTherapy, Trial Assigning IndividuaLized Options for Treatment (Rx), and West German Study Group PlanB, confirm that multigene assays are not interchangeable and that each provides different information for differing patient populations [31].

Composite risk analysis: The composite risk assessment of recurrence risk enables clinicians to estimate the potential advantages of escalating adjuvant ET comprehensively and accurately for premenopausal women diagnosed with HR+/HER2- BC [15]. The composite risk incorporated age, nodal involvement, tumor size, grade, ER, progesterone receptors (PRs), and antigen Kiel 67 (Ki-67) labeling index values. Pagani et al. [15] reported that four or more positive nodes, tumor grade 2 or 3, and younger age (≤40 years) contributed maximally to the multivariable model.

The panel recommended that a patient should be considered to be at high risk of recurrence based on the following criteria: node-positive 1-3, ER/PR <50%, grade 2/3, tumor size of >2 cm, and Ki-67 >20% (statement no. 4) (level of evidence: 2b, 5, 5). This statement concords with the prevailing literature [15,32,33]. In their consensus review, the hIgh RIsk DEfinition in breast cancer working group presented the following risk factors as being associated with a high risk for relapse: nodal status (N2/N3), ER (<10%), PR (<20%), grade (3), tumor size (T 3/4), and Ki-67 (>30%) [32]. The panelists advised that similar data need to be obtained for the Indian scenario to validate these findings.

OFS considerations

Considerations for OFS

ET has been widely acknowledged as an important adjuvant therapy in premenopausal women with HR+ eBC. Numerous studies have demonstrated the efficacy of OFS, tamoxifen, and AI (alone or in combination) in patients with HR+ eBC [48]. The panel (83%) recommended that women with high-risk HR+/HER2- eBC should be considered for OFS in addition to adjuvant oral ET (statement no. 5) (level of evidence: 1, 1, 5, 1). This consensus statement is in agreement with the guidelines of the 2023 St. Gallen International Consensus, the 2018 ASCO, the fifth International Consensus for Breast Cancer in Young Women guidelines, and the Italian Association of Medical Oncology [34-37].

The panel unanimously recommended that OFS should not be offered to all premenopausal patients with HR+/HER2- eBC and that risk stratification should be performed in every patient before they are offered OFS (statement no. 6) (level of evidence: 1a). This statement is supported by a Cochrane review that asserts that the choice of using OFS may be based on tumor and patient characteristics and the side effects of OFS [8].

Duration of OFS

Most (83%) experts recommended that the optimal course of OFS treatment should be up to five years in premenopausal women with HR+/HER2- eBC, if tolerated; otherwise, it can be terminated at two years (statement no. 7) (level of evidence: 5). The 2019 St. Gallen International Consensus Guidelines support the experts’ recommendation. The duration of OFS use in the ASTRRA study was two years, whereas it was five years in the SOFT and TEXT studies. Encouraging findings were reported in both studies, although they differed in patient characteristics and the use of adjuvant CT. Hence, the optimal duration of OFS treatment needs an individualized approach [18].

Choice of Agent

The agents of choice for medical OFS are LHRHa, which includes goserelin, triptorelin, and leuprorelin. The experts agreed that there is no head-to-head comparison between these three drugs and that all three need to be considered similar in terms of efficacy. However, there is a major difference concerning the quality of life associated with their administration. Although the panel acknowledges that all three options have similar efficacies, the panel (92%) still recommended that triptorelin should be preferred over other LHRHa in premenopausal women with HR+/HER2- eBC (statement no. 8) (level of evidence: 2b). The smaller needle size required to administer triptorelin gives it an advantage over the other drugs, as this factor could impact treatment adherence. Triptorelin was the main treatment used to achieve OFS in the SOFT and TEXT trials. It was administered to 91% and 100% of patients in the SOFT and TEXT studies, respectively [14,38].

Frequency of LHRHa

No definitive consensus was attained for either a monthly (50%) or a three-monthly (8%) LHRHa formulation for OFS treatment in premenopausal women with HR+/HER2- eBC [39]. Around 42% of panelists opted for both regimens (statement no.9) (level of evidence: 1b). The panelists discussed and concluded that there is insufficient evidence for preferring monthly or three-monthly regimens and that the choice of treatment can vary according to the country. However, it was pointed out that patients may prefer the three-monthly administration formulation over the monthly administration regimen. However, Bardia and Hurvitz [55] preferred the monthly administration regimen in their review article, citing that there was a lack of adequate evidence on the efficacy of the three-monthly administration regimen.

In a Japanese study, a three‑month goserelin (10.8 mg) treatment demonstrated noninferiority over a monthly (3.6 mg) treatment for progression-free survival at 24 weeks in premenopausal women with ER+ BC. Acknowledging that both regimens possess similar pharmacological properties and safety profiles is imperative [56]. One of the experts suggested that, after monitoring the women’s estradiol levels, the three-monthly routine might be an option for women >40 years of age, especially those who travel from distant locations for treatment. However, a monthly schedule is preferable for women <40 years of age to guarantee sustained OFS. The experts also discussed and concluded that the ESMO guidelines recommend using a three-monthly regimen with close monitoring of ovarian function on a case-by-case basis if monthly use is not feasible or accepted [34].

OFS in Perimenopausal Women

The panel (100%) recommended that OFS should be considered in perimenopausal women with HR+/HER2- eBC if the baseline follicle-stimulating hormone (FSH) and estradiol levels are not in the postmenopausal range (statement no. 10) (level of evidence: 1a) [12]. Serum FSH and estradiol levels help establish menopausal status (statement no. 11) (level of evidence: 5). It was suggested that the recommendations made by the Indian Menopause Society be followed [40]. Markers used in diagnosing menopause are usually restricted for use in special situations or for issues regarding fertility. Levels of FSH >10 international units (IU)/L and >20 IU/L are indicative of declining ovarian function and ovarian insufficiency, respectively, in perimenopausal women with vasomotor symptoms, even if the women are still menstruating. The FSH levels >40 IU/L across tests performed at least four weeks apart are reliable markers for menopause or impending menopause and are associated with low estradiol levels [40].

Bisphosphonates or receptor activator of nuclear factor-kappaB (RANK) inhibitors should be considered in all women who undergo OFS, either as adjuvant therapy or as a preventive strategy (statement no. 12) (level of evidence: 1a) [41]. The experts believed that bisphosphonates or RANK ligand inhibitors should be recommended, especially in women with osteopenia and osteoporosis. However, in premenopausal women, osteopenia or osteoporosis may develop later; therefore, the decision to use RANK inhibitors should be based on the T-score or Z-score. One of the experts suggested a paucity of long-term follow-up trials and indicated that bisphosphonates or RANK inhibitors could be considered as a preventive strategy in patients with low T-scores and as an adjuvant strategy in very high-risk patients. However, while this may prevent osteoporosis and bone metastases, it does not improve overall OS. The meta-analysis by the Early Breast Cancer Trialists’ Collaborative Group recommended adjuvant bisphosphonates therapy for postmenopausal and premenopausal patients with eBC who are receiving OFS [57]. The European panel recommended that bisphosphonates may be considered for routine clinical practice in preventing bone loss induced by cancer treatment in all patients with eBC with either a T-score of ≤2.0 or clinical risk factors of ≥2 for fracture [42]. The panel (91%) recommended that OFS with oral ET may benefit some perimenopausal women with eBC (statement no. 13) (level of evidence: 5).

ET considerations

ET Considerations Along With OFS

The panel (92%) recommended that preference should be given to AIs over tamoxifen as the backbone for OFS (statement no. 14) (level of evidence: 1a, 1b). The experts agreed that the AI of choice is exemestane, which was used in the SOFT and TEXT trials; however, they agreed that no head-to-head comparison between exemestane and other AIs is available. A combined analysis of data from the SOFT and TEXT trials, which compared outcomes in 4690 premenopausal women with ER/PR+ eBC who were randomly assigned to five years of exemestane+OFS with those assigned to tamoxifen+OFS, showed that after a median follow-up of nine years, exemestane+OFS significantly improved disease-free survival (DFS) and distant recurrence-free interval (DRFI), but not OS, compared with tamoxifen+OFS. In the intention-to-treat population, patients who were assigned exemestane+OFS saw significant benefits. The 12-year DFS showed 4.6% absolute improvement (hazard ratio, HR = 0.79; 95% confidence interval, CI = 0.70-0.90; p < 0.001). Additionally, DRFI showed a 1.8% absolute improvement (HR = 0.83; 95% CI = 0.70-0.98; p = 0.03). However, no improvement was observed in OS, which was 90.1% vs. 89.1% (HR = 0.93; 95% CI = 0.78-1.11) [16]. Notably, the advantages appear restricted to patients with intermediate-to-high-risk clinicopathological features and in those with HER2- disease, representing 84% of the trial population [33]. Another meta-analysis of data from 7,030 women from all the available trials on OFS (Austrian Breast and Colorectal Cancer Study Group trial XII, TEXT, SOFT, and Hormonal BOne Effects) reaffirmed that AIs are linked with a reduced risk of BC recurrence compared with tamoxifen [11]. Additionally, the Italian Association of Medical Oncology preferred AIs over tamoxifen [35].

The panel (87%) suggested that tamoxifen monotherapy can be considered in low-risk patients for whom CT is not recommended (statement no. 15) (level of evidence: 1, 5, 5, 1). This statement is supported by the St. Gallen International Consensus Guidelines, as well as the ASCO and NCCN guidelines [33,36,37,43].

The panel (96%) recommended that AIs should not be offered without OFS for premenopausal women (statement no. 16) (level of evidence: 1a). Since premenopausal women secrete higher ovarian estrogens in response to pituitary gonadotropins than postmenopausal women, AIs have a limited capacity to lower estrogens in premenopausal women [44,55].

The panel (96%) recommended that adding AIs, either for five years or for two to three years after tamoxifen treatment for two to three years, in women at intermediate-to-high risk of relapse can decrease the risk of recurrence rather than five years of tamoxifen monotherapy (statement no. 17) (level of evidence: 1b). The experts’ recommendations are based on data from the SOFT and TEXT trials [15], supplemented by clinical experience in selected cases, and literature evidence on the use of AI in postmenopausal women.

CT considerations

CT Considerations and Treatment Strategies

The panel recommended that CT (96%) should be considered in HR+/HER2- eBC with a high risk of recurrence based on clinicopathological assessment and/or genomic assay (statement no. 18) (level of evidence: 1, 5). The NCCN and St. Gallen International Consensus guidelines strongly endorse the value of genomic assays in guiding CT decisions for lymph node-negative disease (Table 6) [6,36]. The NCCN guidelines stratify the recommendations for adjuvant CT based on lymph node metastases (Table 6) [43].

**Table 6:** Systemic adjuvant treatment for HR+/ HER2- disease premenopausal patients with pT1-3 and pN+ tumors CT: chemotherapy; ET: endocrine therapy; HER2-: human epidermal growth factor receptor 2-negative; HR+: hormone receptor-positive; OFS: ovarian function suppression Source: [6,36,43]

Patient type	Metastasis	Decision for CT	Recommended therapy
HR+/HER2- disease premenopausal patients with pT1-3 and pN+ tumors	pN1mi (≤2 mm axillary node metastasis) or pN1 (1-3 positive nodes)	Not a candidate for CT	Adjuvant oral ET + OFS
		If a candidate for CT, consider a gene expression assay to assess the prognosis and CT impact	Adjuvant CT followed by ET or adjuvant oral ET + OFS
	pN2/pN3 (≥4 ipsilateral metastases >2 mm)	Adjuvant CT	Adjuvant CT followed by oral ET + OFS

The panel (80%) favors anthracycline- and taxane-based regimens for CT in patients with HR+/HER2‑ eBC; however, four cycles of a taxane-based regimen can be given for a T2NO disease (statement no. 19) (level of evidence: 5, 1a). The choice is supported by the 2019 St. Gallen International Consensus Guidelines [36]. A patient-level meta-analysis of 100,000 women from 86 randomized trials reported that anthracycline+taxane regimens are most efficacious at reducing BC recurrence and death. Regimens with higher cumulative doses of anthracycline+taxane provided greater benefits [12].

The experts (87%) agreed that the benefit of CT in premenopausal women is due to the direct cytotoxic effect of CT on BC cells and the indirect cytotoxic effect of CT-induced OFS (statement no. 20) (level of evidence: 5). This is in agreement with the literature as the cytotoxic injury to the ovaries and amenorrhea can be persistent and result in infertility, particularly in older premenopausal women [17,44].

The panel (84%) recommended that a case-based decision be arrived at to use either concurrent or sequential adjuvant chemo-ET in patients with HR+ eBC (statement no. 21) (level of evidence: 1b). There is no survival difference when LHRHa is used concurrently or sequentially with CT. The ESMO guidelines advise using the concurrent strategy for patients who want to maintain ovarian function during treatment [44]. The three largest trials (Prevention Of Early Menopause Study, POEMS; Prevention of Menopause Induced by Chemotherapy: a Study in Early Breast Cancer Patients-Gruppo Italiano Mammella 6, PROMISE-GIM6; and Anglo Celtic Group OPTION trials) support the addition of LHRHa to CT to inhibit premature ovarian failure and maintain fertility in premenopausal women [45-47].

In the POEMS trial, 218 of 257 patients were eligible and evaluable (105 in the CT+ goserelin arm and 113 in the CT arm). More patients in the CT+ goserelin arm reported at least one pregnancy than those in the CT arm (five-year cumulative incidence = 23.1% and 12.2%, respectively) [47]. In the PROMISE-GIM6 study, the clinical and tumor characteristics of the 133 patients randomized to CT alone and the 148 patients randomized to CT+ triptorelin were similar. The rate of early menopause was 25.9% in the CT-alone group and 8.9% in the CT+ triptorelin group at 12 months [45]. The Anglo Celtic Group OPTION trial showed that goserelin significantly reduced the risk of premature ovarian insufficiency in women aged ≤40 years, who were treated with CT for eBC; however, the effect of goserelin was not statistically significant in women >40 years [46].

In the TEXT trial, CT was started concurrently with triptorelin, while it was given after CT in the SOFT trial. No difference in outcomes was observed between concurrently and sequentially administering triptorelin. The estimated BC-free interval at four years was 84.8% and 82.9% with concurrent and sequential triptorelin administration, respectively, for women aged <40 years at diagnosis (HR = 1.13, 95% CI = 0.69-1.84) and 90.8% and 91.7%, respectively, for women >40 years at diagnosis (HR = 1.10, 95% CI = 0.57-2.14) [58]. It was observed that older premenopausal women may benefit from a sequential approach, while younger women who are not at high risk for CT-induced amenorrhea and wish to preserve their fertility may consider concurrent initiation of LHRHa alongside CT [50].

OFS treatment algorithm

This treatment algorithm aims to optimize treatment decisions for premenopausal women with HR+/HER2- eBC by utilizing OFS. It represents the culmination of rigorous discussions among esteemed experts and an extensive review of relevant literature. For risk stratification, several crucial factors were considered, including age, tumor size, node status, ER/PR expression, tumor grade, Ki-67 levels, lymphovascular/perineural invasion, and genomic assays. By analyzing these factors, patients are classified into distinct risk categories, namely low, intermediate, and high risk. Subsequently, the algorithm offers tailored treatment recommendations based on each patient’s assigned risk category.

## Conclusions

This paper employed a Delphi methodology to gather insights from BC experts on addressing the ambiguity in treating HR+/HER2- eBC in premenopausal women. It aimed to synthesize expert perspectives into a practical framework that could enhance standard clinical practices. Throughout this process, the panelists leveraged their considerable expertise, drawing from direct experience and extrapolating from primary findings and secondary analyses of randomized clinical trials. As a result, most of the statements under consideration achieved consensus. It was recommended to perform comprehensive risk stratification for premenopausal patients with HR+/HER2- eBC. Age was considered an independent risk factor for women ≤40 years old and a composite risk factor for patients ≥40 years old. The duration of OFS recommended was five years. Although the panel acknowledges similar efficacy among all available options, among the available LHRHas, triptorelin is preferred. It is believed that these recommendations will aid in homogenizing the diagnosis and management and thereby enhance the quality of care for patients with BC in India. Since this consensus is focused on the Indian population, it may not be applicable to the global treatment scenario. However, the outcomes of this consensus may enable individualized and evidence-based approaches for guiding the use of OFS in treating Indian patients with HR+/HER2- eBC.

## Appendices

Appendix 1 

**Table 7:** List of members that participated in an online survey during first round of the modified Delphi process

S. no.	Members	Affiliation
1	Dr. Rajeshwar Singh	Director, Medical Oncology, Paras Hospital, Panchkula, India
2	Dr. Anubha Bharthuar	Head of the Department, Medical Oncology and Hematology, Patel Hospital, Jalandhar, India
3	Dr. Davinder Paul	Head of the Department, Medical Oncology, Fortis Hospital, Ludhiana, India
4	Dr. Syed Nisar	Associate Professor, Department of Medical Oncology, Sher-i-Kashmir Institute of Medical Sciences Srinagar, India
5	Dr. Ketan Dang	Consultant, Medical Oncology, Fortis Hospital, Mohali, India
6	Dr. P. Guhan	Director and Consultant, Medical Oncologist, Sri Ramakrishna Hospital, Coimbatore, India
7	Dr. Bharath Rangarajan	Consultant, Medical Oncologist, Kovai Medical Center and Hospital, Coimbatore, India
8	Dr. P. N. Satyamoorthy	Consultant, Medical Oncologist, Avadi Cancer Clinic, Chennai, India
9	Dr. Jyosthna Elangandula	Consultant, Medical Oncologist, Krishna Institute of Medical Sciences Hospital, Hyderabad, India
10	Dr. Rakesh Pinniti	Consultant, Medical Oncologist, Basavatarakam Indo American Cancer Hospital and Research Institute, Hyderabad, India
11	Dr. Adwaita Gore	Associate Director, Medical Oncology, Nanavati Max Super Speciality Hospital, Mumbai, India
12	Dr. Ashish Joshi	Consultant, Medical Oncologist, Nanavati Max Super Speciality Hospital, Mumbai, India
13	Dr. Tushar Patil	Consultant, Medical Oncology, Sahayadri Hospital, Pune, India
14	Dr. Shruti Kate	Consultant, Medical Oncology, Healthcare Global Enterprises Ltd. Manavata Curie, Nashik, India
15	Dr. Salil Patkar	Director, Oncura Hematology and Oncology Care, Navi Mumbai, India
16	Dr. Tejinder Singh	Senior Consultant Medical Oncologist, Apollo Hospital, Navi Mumbai, India
17	Dr. Chandreshekhar Pethe	Senior Consultant Medical Oncologist, Mumbai Oncocare Center Cancer Care and Research Centre, Nashik, India
18	Dr. Bhushan Nemade	Consultant Clinical Oncologist, Sankalp Speciality Hospital, Nashik, India
19	Dr. Anup Toshniwal	Consultant Medical Oncologist, Medicover Hospital, Aurangabad, India
20	Dr. Varun Nagori	Consultant Medical Oncologist, Marathwada Cancer Hospital and Research Institute, Aurangabad, India
21	Dr. Ashish Bakshi	Consultant Medical Oncologist, Lakhumal Hiranand Hiranandani Hospital, Mumbai, India
22	Dr. Bhuvan Chugh	Senior Consultant, Medical Oncology, Max Superspeciality Hospital, Saket, New Delhi, India
23	Dr. Priya Tiwari	Unit Head, Medical Oncology, Artemis Hospital, Gurgaon, India
24	Dr. Devavrat Arya	Senior Director, Medical Oncology, Max Superspeciality Hospital, Saket, New Delhi, India
25	Dr. Pankaj Goyal	Senior Consultant, Medical Oncology, Rajiv Gandhi Cancer Institute and Research Centre, New Delhi, India
26	Dr. Akhil Kapoor	Associate Professor, Mahamana Pandit Madan Mohan Malviya Cancer Centre, Varanasi, India
27	Dr. Alok Gupta	Director, Department of Medical Oncology, Medanta Hospital, Lucknow, India
28	Dr. P. N. Mohapatra	Head of the Department, Medical Oncology, Apollo Hospital, Kolkata, India
29	Dr. Joydeep Ghosh	Senior Consultant, Medical Oncology, Apollo Hospital, Kolkata, India
30	Dr. Indranil Ghosh	Senior Consultant, Medical Oncology, Apollo Hospital, Kolkata, India
31	Dr. Sandeep Ganguly	Senior Consultant, Medical Oncology, Apollo Hospital, Kolkata, India
32	Dr. Saurav Mishra	Head of the Department, Department of Medical Oncology, All India Institute of Medical Sciences Bhubaneswar, India
33	Dr. M. V. Chandrakant	Senior Consultant, Medical Oncology, Narayana Multispecialty Hospital, Howrah, India
34	Dr. Amit Kumar	Consultant Medical Oncologist, Medanta Hospital, Patna, India
35	Dr. Arnab Bhattacharya	Consultant Medical Oncologist, Tata Medical Centre, Kolkata, India
36	Dr. Sushant Pai	Consultant Medical Oncologist, HCG Hospital, Cuttack, India
37	Dr. Sanyo P. Dsouza	Consultant Medical Oncologist, Kasturba Medical College Hospital, Mangalore, India
38	Dr. Prashant Parmeswaran	Senior Consultant, Medical Oncology, Melathu Veettil Raghavan Cancer Centre and Research Institute, Calicut, India
39	Dr. Narayanankutty Warrier	Medical Director, Melathu Veettil Raghavan Cancer Centre and Research Institute, Calicut, India
40	Dr. Arun Chandrasekharan	Consultant Medical Oncologist, Aster Malabar Institute of Medical Sciences, Calicut, India
41	Dr. Arun R. Warrier	Senior Consultant Medical Oncologist, Aster Medicity, Cochin, India
42	Dr. Poonam Patil	Consultant Medical Oncologist, Manipal Hospitals, Bengaluru, India
43	Dr Rahul Jaiswal	Consultant Medical Oncologist, Hemato Oncology Clinic, Vedanta, Ahmedabad, India
44	Dr. Abhishek Kakroo	Consultant Medical Oncologist, Hemato Oncology Clinic, Vedanta, Ahmedabad, India
45	Dr. Chintan Shah	Consultant Medical Oncologist, Hemato Oncology Clinic, Vedanta, Ahmedabad, India
46	Dr. Anshul Agarwal	Consultant Medical Oncologist, Aviva Oncology Centre, Vadodara, India
47	Dr. Rajesh Patidar	Consultant Medical Oncologist, Choithram Hospital and Research Centre, Indore, India
48	Dr. Dinky Gajiwala	Consultant Medical Oncologist, Apollo Cancer Centre, Navsari, India
49	Dr. Vikas Asati	Consultant Medical Oncologist, Choithram Hospital and Research Centre, Indore, India

Appendix 2 

**Table 8:** List of steering committee members second round of the modified Delphi process HOD: head of the department; HOC: Hemato Oncology Clinic

Members	Affiliation
Prof. Dr. Olivia Pagani	Interdisciplinary Cancer Service, Hôpital Riviera-Chablais, Rennaz, Vaud; Faculty of Medicine, University of Geneva; Faculty of Medicine, University of Lugano, Lugano
Dr. Ashok Kumar Vaid	Chairman, Medical Oncology and Hematology, Medanta Cancer Institute, Gurugram
Dr. Sripada Venkata Sesha Satyanarayana Prasad	Senior Consultant, Medical Oncologist, Apollo Hospital, Hyderabad
Dr. Anubha Bharthuar	HOD Medical Oncology and Hematology, Patel Hospital, Jalandhar
Dr. Vijay Agarwal	Senior Consultant, Medical Oncologist, Apollo Hospital, Bangalore
Dr. Prabrajya Narayan Mohapatra	HOD Medical Oncology, Apollo Hospital, Kolkata
Dr. Chirag Desai	Senior Consultant, Director Medical Oncology, HOC Vedanta Hospital, Ahmedabad
Dr. Jyoti Wadhwa	Vice Chairperson, HOD Medical Oncology and Hematology, Paras Healthcare, Gurugram
Dr. Tarini Prasad Sahoo	Senior Consultant, Medical Oncology, Silverline Hospital, Bhopal
Dr. Ghanshyam Biswas	Consultant Medical Oncology and Director, Sparsh Hospital, Bhubaneswar, Odisha
Dr. Seema Gulia	Consultant, Tata Memorial Hospital, Mumbai
Dr. Anita Ramesh	Senior Consultant, Kauvery Hospital, Chennai
